# Primary Prevention Programme for Burnout-Endangered Teachers: Follow-Up Effectiveness of a Combined Group and Individual Intervention of AFA Breathing Therapy

**DOI:** 10.1155/2013/798260

**Published:** 2013-08-28

**Authors:** Katja Goetz, Thomas Loew, Regina Hornung, Laura Cojocaru, Claas Lahmann, Karin Tritt

**Affiliations:** ^1^Department of General Practice and Health Services Research, University Hospital of Heidelberg, 69115 Heidelberg, Germany; ^2^Department of Psychosomatic Medicine, University Hospital of Regensburg, 93053 Regensburg, Germany; ^3^Administration of Research, AFA Arbeits- und Forschungsgemeinschaft für Atempflege e.V. Berlin, 10823 Berlin, Germany; ^4^Department of Psychosomatic Medicine, University Hospital on the Right of the River Isar, Munich, 81675 Munich, Germany

## Abstract

*Background*. Early retirement of teachers due to burnout is frequent in Germany. In this study short- and medium-term effects of AFA breathing therapy were evaluated. *Methods*. This study was designed as a longitudinal controlled intervention design with four points of measurements: before assessment (T1), after intervention (T2), three months (follow up 1) (T3) after intervention, and six months (follow up 2) after intervention (T4). The intervention lasted a total of 11 weeks (weekly group therapy for eight weeks and three weeks of individual breathing session). The effects of intervention were measured with the questionnaire “work-related behaviour and experience Patterns” (AVEM) at four times. *Results*. In the intervention group 64 teachers and in the self-selected control group 27 teachers were included. The AVEM scales “subjective significance of work” and “professional ambition” changed over time and within both groups (interaction effect). Significant improvements over the four measurements were observed in the intervention group in two AVEM scales: “emotional distancing” (*F* = 6.3; *P* < 0.01) and “balance and mental stability” (*F* = 4.4; *P* < 0.02). *Conclusions*. AFA breathing therapy showed short- and medium-term effects in the intervention group over four points of measurements. It may be assumed that breath therapy supports teachers in resisting occupational demand.

## 1. Introduction

In Germany, teachers leave their jobs on average ten years earlier than the legal retirement age of 65 [1]. The main reason for their premature retirement is attributed to stress-related psychosomatic dysfunctions [2, 3]. In addition to depression, stress, or adaptation problems, burnout or exhaustion are also cited as specific work-related risks of illness for teachers [4–7]. The high occurrence of these symptoms in teachers shows that their work can be considered as rather stressful as well as impairing for their wellbeing, making them a high risk group for the development of burnout. These issues have also been examined in empirical studies. Sickness-related early retirement [1, 4, 8, 9] and the severity and extent of occupational stress in teachers [10–12] have been documented sufficiently in multiple studies. 

The reported imbalance between social demands accompanied by stress and resilience can be seen as a possible starting point for their developing burnout [13]. The progression of burnout is described as a slow process, coupled with increasing emotional fatigue, depersonalization, and reduced personal productivity [6, 14]. The affected individuals are assumed to lack flexible coping strategies, personal resources, and adequate social support [5]. As a consequence the actual illness is related to high subjective psychological strain on part of the individuals accompanied by premature retirement. It should also be kept in mind that mental disorders, especially depression and burnout, are associated with enormous economic costs [15].

Therefore, one important aim is the prevention of burnout based on effective measures and therapies. In general, empirical support regarding the efficacy and effectiveness of such prevention measures is scarce. While Awa et al. [16], for example, showed that a wide spectrum of approaches for burnout prophylaxis is available, they regretfully refrain from making statements regarding the efficacy of the different approaches reported. Van Straten et al. [17] showed first empirical results of an innovative strategy to prevent burnout using an internet-based treatment manual conceptualized as a self-help intervention to reduce work-related stress. Another possible strategy to prevent burnout could be breath therapy. Breath therapy has been viewed capable of affecting the psychosomatic structure and causing positive psychosomatic changes [18]. 

The intention of this study is to evaluate the effects of a specific breath therapy, the AFA breathing therapy, as an approach for primary prevention of burnout in teachers. AFA breathing therapy has a focus on the perception of individual breathing, which is taken as a starting point for prophylaxis. This approach differs fundamentally from medical, symptom-related breathing therapy. Due to the holistic approach AFA breathing therapy is regarded as helpful for different general problems, such as breathing issues, posture problems, coping with stress, preservation of productivity (also as a self-help measure), for strengthening self-competence, and for working on breathing and voice in strongly communicative professions (such as singers and musicians). In general, the treatment aims at promoting health, wellbeing, and resistance against stress or burnout risk [19].

To our knowledge research to breath therapy is rare. Therefore, in our study a combination of group and individual therapeutic intervention of AFA breathing therapy was performed to identify possible influences of different settings. The objective of this study was to evaluate short- and medium-term effects of breath therapy on the work-related and experience patterns of teachers.

## 2. Materials and Methods

This study was designed as a longitudinal controlled intervention design with four points of measurements: before assessment (T1), after intervention (T2), three months (follow up 1) (T3) after intervention, and six months (follow up 2) after intervention (T4). 

### 2.1. Recruitment

**Figure 1:**
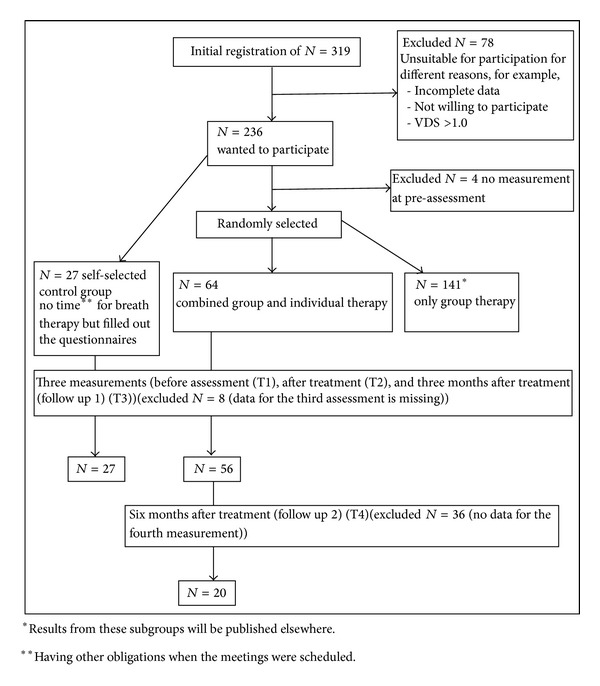


At the beginning of the school year 2005/2006, teachers were recruited in cooperation with the Berlin Senate Department for Education, Youth and Sports and the Bavarian State Department for Education and Cultural Affairs. Flyers were distributed in the regional schools seeking volunteers. After signing an informed consent form, the potential candidates could register using an alias. A total of 319 teachers showed initial interest and registered online. Thereof, 27 refused to take part in the intervention group but were willing to complete questionnaires (self-selected control group) before assessment (T1), after intervention (T2), and at three months (follow up 1) (T3) after intervention. The remaining 209 teachers were randomly allocated to different intervention groups: breath therapy in individual and group setting (*n* = 64) and breath therapy in group setting only (*n* = 141), respectively. Four teachers had to be excluded from analysis because of missing preassessment data. As an inclusion criterion, only subjects not suffering from a diagnosable mental illness were allowed to participate in the study. This was determined by using the VDS 90 (Behavioural Diagnostic and Therapy Planning Manual) [20], a screening instrument designed for detecting mental disorders in accordance with ICD-10. Subjects with a total VDS 90 score of ≤1.0 were included in the study. 64 of the registered teachers were assigned to a combination of group and individual therapeutic intervention using AFA breathing therapy. The intervention lasted a total of 11 weeks. This included weekly group therapy for eight weeks, followed by three weeks of individual breathing sessions once a week. The remaining participants took part in other study arms. Participants did not receive any fee for their participation in the study. Detail information is shown in Figure 1.

### 2.2. Intervention

AFA breath therapy was developed in 1958 with participation of different health care professionals (medical and nonmedical professionals) in Germany. Therapists are organised in a professional organisation called “Berufsverband für Atempädagogik und Atemtherapie e.V.” It is possible for therapists to receive an AFA Diplom. 

According to AFA breathing therapy/training aims are at approaching the individual as a whole. Listening to your breath and experiencing and permitting its natural flow continuously are an essential part of the therapy, which includes perceiving and experiencing the body during movement and calm relaxation. Breathing should not be manipulated through certain techniques but should follow its natural rhythm. Using continuous mindfulness, breathing develops into a source of energy. Blockades and muscle hardening are noticed and can loosen up; the space around the soul is freed up [21]. 

AFA Breathing Therapy was given on the basis of a 16-page standardised manual by a total of 12 AFA breathing therapists (10 women and 2 men), who had all completed AFA training. All therapists had a work experience of more than 2 years and worked in their own practices. To minimize the effect of different therapists a standardised manual was used. The manual was based on different elements such as stretching and pressure, motion sequence, breath space work, and source of tension within breath. The breath therapy exercises are repetitive, and the relationship of body movement and patterns of breath regarding teachers working situation would be an important experience to prevent burnout. The awareness of the sensation of the movement of breath throughout the body is regarded as the basis of breath experience process and the basis of each exercise within the manual.

### 2.3. Measurements

The assessments were performed using “psychopathology screening” (VDS90) and a self-assessment questionnaire on “work-related behaviour and experience patterns” (AVEM). 

VDS90 is a psychological research tool for screening mental health problems based on ICD-10 [20]. This self-assessment tool, designed for outpatient psychotherapy, consists of 90 items. Based on a factor-analytic study, 26 different mental health problems are assessed, which reflects the patient's symptoms comprehensively and is essential for a syndrome diagnosis of mental problems, showing high internal consistency (Cronbach's alpha: 0.93) for the total VDS90 score. This instrument was used for rating the severity of mental and psychosomatic issues of patients [20]. The VDS90 score ranged between 0 (no mental health problem) and 3 (different mental health problems). 

The questionnaire “work-related behaviour and experience patterns” (AVEM) is used for a quantitative evaluation of the subjective perception of work-related behaviour and experience patterns and is a well-known questionnaire in international publications [22–25]. It includes a total of 66 items which were summarized to 11 dimensions/scales based on factor analysis. See Table 2 for description of the 11 scales/dimensions. The dimensions show very good to good internal consistency (Cronbach's alpha: 0.78–0.87). According to burnout research, professions with increased psychological stress are often characterised by an enormous impairment of subjective wellbeing and specific work-related performance [6, 22, 26]. AVEM does not only assess professional commitment but also resistance to stress and work-accompanying emotions [21]. This questionnaire was specifically constructed for research evaluating issues concerning the psychology of work and health. In contrast to Maslach burnout inventory developed by Maslach et al. [27], AVEM evaluates a broad range of occupational demands and aimed to support individual resources. Furthermore, the AVEM seems important for intervention related diagnostic of occupational coping behaviour. 

### 2.4. Data Analysis

Data evaluation was undertaken with the statistics programme SPSS (version 20.0). Differences between control and intervention groups were analysed using Student's *t* test for continuous variables as appropriate and Chi-square test for categorical variables. Furthermore, a variance analyses for repeated measurements were performed. At first, the control and intervention groups were compared considering the time and interaction (time × group) effect over three measurement points. In addition, the intervention group was compared considering the time effect over four measurement points. *P* ≤ 0.05 was determined as the significance level.

## 3. Results

The sociodemographic data of intervention and control groups is listed in Table 1. Both groups were equally distributed regarding the sociodemographic variables. 

### 3.1. Longitudinal Effects of Breath Therapy in Intervention and Control Group

The longitudinal effects of breath therapy in intervention and control group are presented in Table 2. The variance analyses for repeated measurement showed in some dimensions a time effect and a time × group effect as well. The dimensions “subjective significance of work” and “professional ambition” showed a time effect and time × group effect in favour of the control group. A nonsignificant tendency was observed in the dimension “balance and mental stability” in favour of the intervention group. Furthermore, a significant time effect in the intervention group was observed in the dimension “tendency to exert” showing an improvement. 

### 3.2. Longitudinal Effects of Breath Therapy

Analysing longitudinal effects, only subjects who had completed all measurements—four measurements in the intervention group (*n* = 20) and three measurements in the control group (*n* = 27)—were included in the variance analyses of repeated measurements. In the intervention group (Table 3) significant improvements over four points of measurements were observed in the dimensions “emotional distancing” (*F* = 6.3; *P* < 0.01) and “balance and mental stability” (*F* = 4.4; *P* < 0.02). Furthermore, nonsignificant tendencies were evaluated within the dimensions of “tendency to exert” (*F* = 3.0; *P* = 0.06), “experience of social support” (*F* = 2.7; *P* = 0.08), and “offensive coping with problems” (*F* = 2.6; *P* = 0.09). 

With exception of “subjective significance of work” (*F* = 21.5; *P* < 0.01) and “professional ambition” (*F* = 9.6; *P* < 0.01) no improvements were observed in the control group over three points of measurements (Table 4).

## 4. Discussion

In teachers receiving AFA breathing therapy in a combined individual and group setting, positive changes in work-related and experience patterns could be observed indicating a possible effect as a primary prevention strategy. However, also in the control group significant improvements were observed during the same period of time.

 The following results were evaluated regarding the work-related and experience patterns of teachers. 

The intervention group showed improvements on two of the 11 dimensions between four points of measurements: “emotional distancing” and “balance and mental stability”. The dimensions “tendency to exert,” “offensive coping with problems,” and “experience of social support” revealed a statistical tendency in the assumed direction. In the control group, the variance analyses with repeated measurements over three time points showed a significant worsening in the control group for the dimensions “subjective significance of work” and “professional ambition”. In view of these undesired changes in the control group, it could be argued that the intervention might have prevented a similar development in the intervention group on those two scales. 

In summary, the subjects' mean scores showed stability or significant improvement on a variety of AVEM scales from preassessment one up to the follow up one after six months. In particular the follow up, taken six months after the end of the intervention, may be interpreted that AFA breathing therapy could positively affect work-related experience and behaviour patterns on an intermediate range. In particular the scales “emotional distancing” and “balance and mental stability” showed significant improvements. Resistance to occupational demands could have an essential impact to avoid a burnout [28]. 

There is a growing body of evidence pertaining to AFA breathing therapy. In a study of teachers and managers in psychosomatic rehabilitation, Beutel et al. [29] reported positive results for AFA breathing therapy similar to those of this study. Viewing individual comparisons of the AVEM scales, the greatest differences occurred between the preassessment one and follow up one, a finding which is confirmed by this study. 

The effectiveness of AFA breathing therapy in specific patient groups was the subject of two further studies [29, 30]. However, the findings regarding the measure's effectiveness are not conclusive. In one study, the positive effects on patients with chronic back problems are based merely on therapist statements [31]. In the second study, no statistically significant changes could be found for patients with bronchial asthma following breathing therapy, which could be attributed to the small sample size of the study [32]. Overall, it has been reported that mind-body therapies as breath therapy can have a positive effect on patients well-being [33]. Moreover, Esch et al. showed in a pilot study that mind-body techniques could reduce physiological and psychological stress which should reviewed in further intervention studies with breath therapy [34]. It could be assumed that self-care activities are an important treatment concept for the prevention of burnout.

Despite the positive results reported here, our study has some limitations. Possible effects of sample attrition, which cannot be assessed with the available data, should be taken into account. Furthermore, our study is a small pilot study with an explorative design. However, to be able to detect strong effects with an 80% probability 70 participants would have to be analysed in each group. 

A replication of this study with a larger sample size (intervention and control samples) could help shed light on these matters. In addition, in view of the voluntary nature of participation in this study, which required a certain amount of motivational readiness on part of the subjects, we cannot generalize the results to all types of teachers or to individuals with other professions. Moreover, we have not assessed further demographic characteristics of the participating teachers sociodemographic information of teachers such as comorbidities; length and amount of employment should be assessed in further studies and evaluated as covariates. To further study the efficacy and effectiveness of AFA breathing therapy, it would be, of course, interesting to find out for which group of teachers this treatment proves itself most effective and which factors, like interest and/or motivation, promote access to the approach. Furthermore, such analysis could help determine important indicators which could be used to spread the method more effectively. At the same time such results could improve the evaluation of the generalizability of our results. It would also be interesting to know if the participants of the study keep up the practice of AFA breathing therapy after the intervention ending and integrate it into their everyday lives. The effects of this variable could prove itself relevant for the interpretation of follow up effects. 

In spite of the mentioned limitations, this study could provide first insights into a possible strategy to prevent burnout by using AFA breathing therapy. Furthermore, our study provides useful information regarding recruitment, kind of therapy, and effects sizes to plan further large-scale studies on this issue. 

## Figures and Tables

**Table 1:** Sociodemographic characteristics.

Characteristics	Intervention group (*N* = 68)		Control group (*N* = 27)	
	Mean	SD	Mean	SD
Age (in years)	47.3	7.2	47.5	7.4

	*N*	%	*N*	%

Sex				
Female	55	80.9	22	81.5
Male	13	19.1	5	18.5

SD: Standard deviation; *N*: number.

**Table 2:** Longitudinal effects of breath therapy in intervention and control groups.

AVEM Scale**		Intervention group (*N* = 56) Mean (SE)	Control group (*N* = 27) Mean (SE)	Time effect *F* (*P*-value)	Time × group effect *F* (*P*-value*)
(1) Subjective significance of work	T1	15.0 (0.6)	14.7 (0.9)	36.6 (<0.01)	40.7 (<0.01)
	T2	14.8 (0.6)	21.6 (0.9)		
	T3	15.0 (0.6)	14.6 (0.9)		

(2) Professional ambition	T1	17.0 (0.6)	17.1 (0.9)	17.8 (<0.01)	15.6 (<0.01)
	T2	16.9 (0.5)	21.9 (0.8)		
	T3	16.6 (0.6)	17.0 (0.9)		

(3) Tendency to exert	T1	21.3 (0.5)	20.6 (0.8)	4.7 (0.01)	0.4 (0.7)
	T2	20.3 (0.6)	19.9 (0.9)		
	T3	20.8 (0.6)	19.9 (0.9)		

(4) Striving for perfection	T1	21.7 (0.6)	19.9 (0.9)	2.5 (0.09)	0.4 (0.7)
	T2	21.4 (0.7)	19.1 (1.0)		
	T3	21.2 (0.7)	19.1 (1.0)		

(5) Emotional distancing	T1	14.2 (0.6)	15.1 (0.8)	0.4 (0.7)	0.7 (0.5)
	T2	14.9 (0.6)	15.0 (0.8)		
	T3	14.9 (0.6)	15.0 (0.8)		

(6) Resignation tendencies	T1	17.8 (0.6)	17.3 (0.9)	1.0 (0.36)	1.5 (0.24)
	T2	17.4 (0.6)	17.4 (0.9)		
	T3	16.9 (0.6)	17.4 (0.9)		

(7) Offensive coping with problems	T1	19.9 (0.5)	19.9 (0.7)	0.3 (0.72)	1.0 (0.38)
	T2	20.4 (0.5)	19.6 (0.7)		
	T3	20.1 (0.5)	19.6 (0.7)		

(8) Balance and mental stability	T1	18.0 (0.6)	17.1 (0.9)	0.3 (0.75)	3.0 (0.06)
	T2	18.9 (0.6)	16.6 (0.9)		
	T3	19.0 (0.6)	16.6 (0.9)		

(9) Satisfaction with work	T1	21.7 (0.6)	22.0 (0.8)	0.28 (0.75)	0.19 (0.83)
	T2	22.1 (0.6)	22.0 (0.8)		
	T3	22.0 (0.6)	22.0 (0.9)		

(10) Satisfaction with life	T1	21.1 (0.6)	21.6 (0.9)	0.3 (0.77)	1.9 (0.16)
	T2	22.0 (0.6)	21.5 (0.9)		
	T3	21.6 (0.6)	21.5 (0.9)		

(11) Experience of social support	T1	20.0 (0.7)	21.9 (1.0)	0.5 (0.63)	1.8 (0.17)
	T2	20.6 (0.7)	21.3 (0.9)		
	T3	19.9 (0.7)	21.3 (1.0)		

SE: standard error.

*Statistical significance *P* < 0.05; **possible score for each scale between 6 (fully agree) and 30 (fully disagree).

**Table 3:** Longitudinal effects of breath therapy in the intervention group.

AVEM Scale**	Intervention group (*n* = 20)				
	T1 Mean (SE)	T2 Mean (SE)	T3 Mean (SE)	T4 Mean (SE)	Time effect *F* (*P*-value*)
(1) Subjective significance of work	14.3 (0.96)	14.1 (0.88)	14.7 (0.85)	14.4 (0.96)	0.65 (0.59)
(2) Professional ambition	16.0 (0.73)	16.7 (0.93)	16.2 (0.82)	16.5 (1.04)	0.58 (0.64)
(3) Tendency to exert	20.2 (0.98)	18.8 (1.03)	18.7 (1.1)	18.7 (1.1)	3.0 (0.06)
(4) Striving for perfection	22.4 (1.1)	21.6 (1.0)	21.4 (1.1)	21.0 (1.1)	1.1 (0.37)
(5) Emotional distancing	13.9 (0.80)	15.6 (0.94)	15.4 (0.85)	16.5 (0.89)	6.3 (<0.01)
(6) Resignation tendencies	18.1 (0.9)	17.1 (1.1)	16.7 (1.1)	16.4 (1.2)	1.2 (0.33)
(7) Offensive coping with problems	19.7 (0.7)	21.1 (0.6)	20.8 (0.6)	21.4 (0.7)	2.6 (0.09)
(8) Balance and mental stability	18.3 (1.0)	19.8 (1.0)	19.5 (1.1)	21.0 (0.9)	4.4 (0.02)
(9) Satisfaction with work	21.7 (0.8)	22.0 (1.0)	22.2 (0.9)	22.7 (0.9)	1.9 (0.17)
(10) Satisfaction with life	21.5 (1.0)	22.9 (0.9)	22.9 (0.9)	23.3 (0.9)	2.1 (0.13)
(11) Experience of social support	21.0 (1.1)	22.0 (1.0)	21.6 (1.1)	23.1 (1.1)	2.7 (0.08)

SE: standard error.

*Statistical significance *P* < 0.05; **possible score for each scale between 6 (fully agree) and 30 (fully disagree).

**Table 4:** Longitudinal effects in the control group.

AVEM Scale**	Control group (*n* = 27)			
	T1 Mean (SE)	T2 Mean (SE)	T3 Mean (SE)	Time effect *F* (*P*-value*)
(1) Subjective significance of work	14.7 (0.81)	21.6 (0.88)	14.7 (0.85)	21.5 (<0.01)
(2) Professional ambition	17.1 (1.01)	21.9 (0.75)	17.0 (0.96)	9.6 (0.01)
(3) Tendency to exert	20.6 (0.72)	19.9 (0.91)	19.9 (0.91)	2.3 (0.14)
(4) Striving for perfection	19.9 (0.83)	19.1 (1.02)	19.1 (1.02)	3.9 (0.06)
(5) Emotional distancing	15.1 (0.84)	15.0 (0.91)	15.0 (0.91)	0.02 (0.90)
(6) Resignation tendencies	17.3 (0.98)	17.4 (1.07)	17.4 (1.07)	0.04 (0.85)
(7) Offensive coping with problems	19.9 (0.78)	19.6 (0.81)	19.6 (0.81)	0.19 (0.67)
(8) Balance and mental stability	17.1 (1.11)	16.6 (1.00)	16.6 (1.00)	1.01 (0.32)
(9) Satisfaction with work	22.0 (0.97)	22.1 (0.88)	22.1 (0.88)	0.01 (0.94)
(10) Satisfaction with life	21.6 (0.82)	21.1 (0.87)	21.1 (0.87)	0.8 (0.37)
(11) Experience of social support	21.9 (0.75)	21.3 (0.69)	21.3 (0.69)	0.8 (0.38)

SE: standard error.

*Statistical significance *P* < 0.05; **possible score for each scale between 6 (fully agree) and 30 (fully disagree).
